# Identifying the unmet health needs of patients with congenital hypogonadotropic hypogonadism using a web-based needs assessment: implications for online interventions and peer-to-peer support

**DOI:** 10.1186/1750-1172-9-83

**Published:** 2014-06-11

**Authors:** Andrew A Dwyer, Richard Quinton, Diane Morin, Nelly Pitteloud

**Affiliations:** 1 University of Lausanne, Institut universitaire de formation et de recherche en soins and the Endocrinology, Diabetes & Metabolism Service of the Centre Hospitalier Universitaire Vaudois, 46 Rue du Bugnon, BH19.317, Lausanne CH-1011, Switzerland; 2 University of Newcastle-upon-Tyne, Institute of Genetic Medicine and the Royal Victoria Infirmary, Newcastle-upon-Tyne NE1 3BZ, UK; 3 University of Lausanne Institut universitaire de formation et de recherche en soins, Biopole 2 - Route de la Corniche 10, Lausanne 1010, Switzerland; 4 University of Lausanne and the Endocrinology, Diabetes & Metabolism Service of the Centre Hospitalier Universitaire Vaudois, 46 Rue du Bugnon, Lausanne 1011, Switzerland

**Keywords:** Congenital hypogonadotropic hypogonadism, Kallmann syndrome, Community based participatory research, Internet, E-health, Rare diseases, Health promotion, Patient-centered care, Nursing

## Abstract

**Background:**

Patients with rare diseases such as congenital hypogonadotropic hypogonadism (CHH) are dispersed, often challenged to find specialized care and face other health disparities. The internet has the potential to reach a wide audience of rare disease patients and can help connect patients and specialists. Therefore, this study aimed to: (i) determine if web-based platforms could be effectively used to conduct an online needs assessment of dispersed CHH patients; (ii) identify the unmet health and informational needs of CHH patients and (iii) assess patient acceptability regarding patient-centered, web-based interventions to bridge shortfalls in care.

**Methods:**

A sequential mixed-methods design was used: first, an online survey was conducted to evaluate health promoting behavior and identify unmet health and informational needs of CHH men. Subsequently, patient focus groups were held to explore specific patient-identified targets for care and to examine the acceptability of possible online interventions. Descriptive statistics and thematic qualitative analyses were used.

**Results:**

105 male participants completed the online survey (mean age 37 ± 11, range 19–66 years) representing a spectrum of patients across a broad socioeconomic range and all but one subject had adequate healthcare literacy. The survey revealed periods of non-adherence to treatment (34/93, 37%) and gaps in healthcare (36/87, 41%) exceeding one year. Patient focus groups identified lasting psychological effects related to feelings of isolation, shame and body-image concerns. Survey respondents were active internet users, nearly all had sought CHH information online (101/105, 96%), and they rated the internet, healthcare providers, and online community as equally important CHH information sources. Focus group participants were overwhelmingly positive regarding online interventions/support with links to reach expert healthcare providers and for peer-to-peer support.

**Conclusion:**

The web-based needs assessment was an effective way to reach dispersed CHH patients. These individuals often have long gaps in care and struggle with the psychosocial sequelae of CHH. They are highly motivated internet users seeking information and tapping into online communities and are receptive to novel web-based interventions addressing their unmet needs.

## Background

Patients affected by rare diseases are dispersed and face a variety of challenges including lack of specialized care, delays in diagnosis, negative social consequences and other psychosocial burdens. The European Organization of Rare Diseases has previously reported a variety of obstacles these patients face [1]. Many of these are critical social determinants of health that place rare disease patients in the realm of health disparities [2]. Further, beyond disease and symptom burden, the rarity of the condition can result in patients and families feeling marginalized. In addition, the psychosocial impact of perceived invisibility, isolation, and feelings of powerlessness can have significant deleterious impact on quality of life [3-5]. One way that patients and families have overcome this is by embracing technology to access information and connect with other patients online [6,7]. Advances in information technology and communications are creating cultural shifts and are changing how people develop expertise. These trends have important implications for healthcare systems and particular relevance for empowering patients dealing with rare diseases [8].

Congenital hypogonadotropic hypogonadism (CHH, ORPHA174590) is a rare, genetic, endocrine disorder which is clinically characterized by incomplete/absent puberty and infertility as a result of a deficiency of gonadotropin releasing hormone. When it occurs with an absent sense of smell it is termed Kallmann syndrome (KS, ORPHA478). While incidence is difficult to assess, it is estimated at one in 4,000-10,000 based on a study of French conscripts [9]. Additionally, it is 2–5 times less frequent in females than in males though this gender discordance may represent a bias of ascertainment [10,11]. Most cases are sporadic, consistent with a condition which impairs fertility, yet approximately a third of cases display a familial pattern of inheritance. CHH is clinically heterogeneous and may occur with variable reproductive (e.g. undescended testes with/without micropenis) and non-reproductive phenotypes such as eye/ear anomalies (including sensorineural deafness), cleft lip/palate, skeletal anomalies, osteoporosis, metabolic disturbances and renal agenesis [11].

As with other rare disorders, CHH patients often experience delays in diagnosis. For these individuals, the absent sexual development of CHH becomes increasingly apparent as peers and younger siblings advance through puberty while they remain in a preadolescent state. This experience can have significant emotional and psychological consequences - as depicted in an early case series [12] and reiterated in a recent patient account [13]. Indeed, studies of newly diagnosed adolescent males with CHH indicate higher levels of anxiety and depressive symptoms compared to peers [14,15]. However, the long-term impact on CHH patients has not been examined. Unlike many other orphan diseases, there are effective treatments available. Hormone replacement therapy can induce development of sexual characteristics and in most cases, fertility [16-18]. Cases of spontaneous reversal has been documented [19], yet patients typically require lifelong therapy. Importantly, normalizing the serum hormone levels does not completely ameliorate the biopsychosocial effects that CHH patients experience. A study of adolescent CHH patients indicated improved mood after 6-months of treatment, yet significant physical and emotional role difficulties persisted compared to healthy peers [14]. These factors may impede adherence to treatment which is a widely-recognized and significant problem in healthcare, particularly in chronic disease, as an estimated 50% find it challenging to meet prescribed regimens regardless of disease, prognosis, or setting [20]. Patients with chronic conditions provide approximately 95% of their care [21] and thus, adherence problems can have important health ramifications. Non-adherence in men with CHH causes hormone levels plummet and patients become hypogonadal, placing them at risk for osteoporosis, anemia and metabolic problems such as insulin resistance [22-27]. A study examining bone health of a cohort of 26 Finnish patients indicated 35% of patients had non-adherence periods exceeding 5 years [28]. So despite the availability of effective treatments, there are seemingly other issues diminishing CHH patients’ self-care practices. However, we do not know the extent of problems with adherence among the general population of CHH men nor do we understand patients’ perceived barriers to adherence and health promoting behavior.

Therefore, the aim of this study was threefold. First, to examine the utility of web-based platforms for reaching dispersed CHH patients and conducting an online needs assessment. Second, to better define patterns of adherence to treatment and unmet health and informational needs of CHH patients. Third, to identify specific patient-reported targets for care and assess the acceptability of launching patient-centered e-health interventions to enhance self-care and health promoting behavior.

## Methods

**Figure 1:**
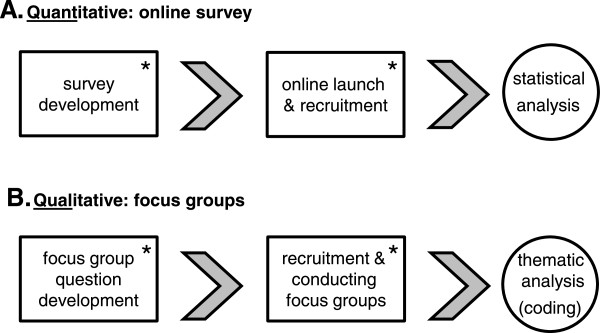
**Study schema.** Schematic depicting the sequential explanatory mixed-methods design. **(A)** First, a quantitative online survey was conducted and statistical analysis performed. **(B)** Subsequently, qualitative focus groups were conducted to explore the survey findings in detail and identify potential explanatory mechanisms. Asterisks note study stages involving participation of patient community leaders.

This study utilized a community based participatory research framework. This approach has previously been put forth as a useful research model for empowering patients and overcoming health inequities [29]. As part of a European network focused on CHH (COST Action BM1105 [30]), partnerships with online patient community leaders (i.e. moderators of online patient support sites) were developed to contribute to the study design, recruitment, and conduct of the study. We recognized patients as experts and these partners were actively involved in generating ideas as well as providing feedback, comments, and criticism in an iterative process to refine the questions and improve the language and clarity of the survey and focus group questions. This descriptive, multivariate correlational needs assessment study employed a sequential mixed-methods design. This approach (QUANT-Qual) started with an online survey and statistical analysis of the quantitative data (Figure 1). Subsequently, patient focus groups were conducted and discussions were analyzed using a qualitative data analysis. The intention behind employing this mixed-methods approach was to provide a deeper exploration of the unmet health needs of CHH men than would be possible by either method in isolation.

Men were targeted for recruitment as CHH is rare and male cases are 2–5 times more common than female cases [10,11]. Men 18–70 years of age diagnosed with CHH [31] were included in the study. A random sampling (40% of subjects) were contacted to confirm diagnosis and those outside of the age range or who had other causes of hypogonadism were excluded from analyses. The study was publicized online via a closed/private CHH social media group (Facebook), CHH forum (chat room), a clinical trials registry [32], and the COST Action website [30]. Focus groups were held in concert with patient-support meetings that were planned jointly by patient community leaders and study investigators.

First, the quantitative arm included an online survey to collect demographic information and to assess healthcare literacy, health information seeking patterns, interactions with healthcare system/providers, and self-reported adherence to treatment/healthcare (Additional file 1). To assess healthcare literacy we used a self-report method previously shown to correspond with lengthier gold standard literacy tests [33,34]. Descriptive statistics, Chi square and Pearson product moment coefficients of correlation were performed on survey results. To evaluate the relative importance of the most frequently used sources of CHH information we performed Kruskall-Wallace one-way analysis of variance on ranks. SigmaStat (Systat Software Inc., San Jose, California, USA) was used for statistical analyses and a p < 0.05 was considered significant.

Second, the qualitative arm involved patient focus groups discussing issues and challenges related to living with CHH, patient-reported coping strategies and the acceptability of possible online interventions. Questions were derived from Pender’s Health Promotion Model [35] and developed with input from patient community leaders. Focus group transcripts were analyzed using NVivo10 (QSR International PSY Ltd., Melbourne Australia). Briefly, thematic analysis (coding) was conducted by two separate investigators (AD:DM) to identify categories of responses and themes emerging from the focus group discussions and discrepancies in coding as well as emergent categories were discussed until resolution was achieved. Iterative coding occurred until no further themes are identified, suggesting a saturation point has been reached. Additionally, connections between coded terms were mapped to examine connections within and between categories (i.e. whether or not certain themes appear together repeatedly) and those arising frequently and expansively were given particular emphasis [36].

The study was approved by the University of Lausanne ethics committee and informed consent was obtained from all participants. Participants in the online survey provided an electronic, opt-in format consent while focus group members provided written consent. All participants received investigator contact information to address questions/concerns and were given the option to provide an email address if they agreed to be contacted by the investigators for follow-up clarification.

## Results and discussion

### Web-based platforms combined with community partnerships are effective for conducting needs assessment survey in dispersed patients

The survey was online for 7-months and received a total of 230 hits. Of these, 105 (46%) were CHH men who completed the survey and met inclusion criteria. To provide context, if one takes the incidence of CHH as 1/10,000, then recruiting 100 subjects would provide an equivalent denominator of 1 million people. Thus, the web-based recruitment was an effective way to reach this dispersed population of CHH men. The sociodemographic information of the men responding to the web-based survey is provided in Table 1. These participants varied in age from 19–66 years (mean 37 ± 11, median 36), two thirds had received education beyond high school/vocational training (69/105, 66%) and the majority were employed (79/104, 76%) across a broad spectrum of fields (Additional file 1). From the relatively advanced age of survey respondents we suspect that younger men with CHH may be lurking online to gather information but may not yet feel emotionally secure enough to discuss issues regarding their lack of sexual development. A review of the email addresses voluntarily provided by participants included email service providers from North and South America, Europe, and Australasia underscoring the global reach of online recruiting via social media for rapidly reaching dispersed patients.

**Table 1:** Sociodemographics of the CHH men completing the online survey (n = 105)

**Age (n = 105)**	**n (%)**
19–29	32 (30%)
30–39	39 (37%)
40–49	19 (18%)
50–59	11 (10%)
60+	4 (4%)
**Education (n = 105)**	
High school/vocational	36 (34%)
University	38 (36%)
Post-Graduate	31 (30%)
**Employment (n = 104)**	
Working full-time	70 (67%)
Working part-time	9 (9%)
Unemployed	10 (10%)
Retired	6 (6%)
Student	9 (9%)
**Relationship status (n = 104)**	
Married	38 (36%)
In a relationship	16 (15%)
Single	25 (24%)
Never been in a relationship	24 (23%)
Divorced	1 (1%)

Studying orphan diseases presents a challenge in terms of recruiting adequate numbers of widely dispersed patients [37,38]. Previously, online web-based questionnaires have proven a useful tool for reaching dispersed populations [39] and advertising on social media platforms can enhance patient recruitment efforts [40,41]. Instead of using online advertising, herein we developed partnerships with online patient community leaders to reach dispersed rare disease patients and utilized web-based platforms for both recruitment and data collection. This was an effective way to reach a large audience and conduct an online CHH needs assessment. Further, such patient community partnerships may provide multiple bi-directional benefits including connecting patients with expert care and enhancing recruitment for research/clinical trials.

### CHH patients have long gaps in care

**Figure 2:**
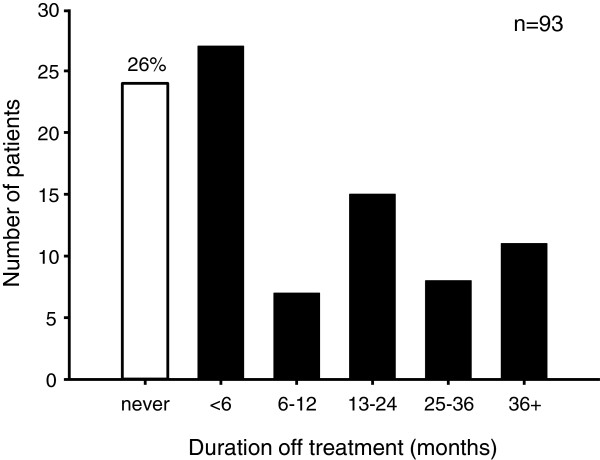
**Adherence to treatment among CHH men.** Patient-reported longest duration off treatment (n = 93). All men had been on treatment for at least 12 months. Only 26% (24/93) of men reported never having a gap in treatment (white bar). In total, 37% (34/93) had a lapse in treatment of more than1 year.

Nearly all the men (99/105, 94%) in the online survey responded that they had received testosterone therapy for CHH. Six men reported never having received testosterone; three men stated they had never been on treatment (2/3 had been recently diagnosed) and the other 3 men had only received gonadotropin injections. In total, only 24/93 (26%) of men reported never having a lapse or gap in treatment (Figure 2). However, 37% (34/93) reported having been off treatment for more than a year. Survey respondents reported similar the periods when asked about the longest duration without healthcare (Additional file 1). No correlation was observed between medication/healthcare adherence and either age of diagnosis or lifetime duration of treatment. Importantly, these gaps in treatment and care can have major health impacts on patients. Hypogonadism causes decreased libido, impaired sexual function and anemia and can have profound effects on bone density placing patients at increased risk for osteoporosis and fracture [22,28]. Further, several meta-analyses point to an association between low serum testosterone levels and the metabolic syndrome and diabetes [24-26]. Even acute withdrawal of treatment in these men induces increased fasting insulin levels and insulin resistance within two weeks [27] while long-term hypogonadism carries heightened prevalence of hyperinsulinemia, metabolic syndrome and diabetes [42]. Thus, lack of or inadequate androgen replacement may heighten further health risks for CHH men such as cardiovascular morbidity. Accordingly, these represent meaningful targets for interventions to enhance adherence to treatment and self-management.

### CHH patients have unmet psychosocial health needs

The online survey included several questions that were developed to examine past interactions with healthcare systems (Additional file 1) including experiences with discrimination related to their condition [43]. Fewer than one in five (16/105, 15%) of men reported that they had faced this type of discrimination. Notably, approximately half of the respondents (54/105, 51%) had been seen at a specialized/academic medical center. As healthcare providers exert important interpersonal influences on health promoting behavior, we queried patients about their experiences with healthcare professionals. In total, 70/105 (67%) stated that their healthcare provider understood the medical aspects of their condition. However, significantly fewer (39/104, 38%, p < 0.001) perceived that their provider understood the emotional impact of CHH. Having a provider who comprehended the medical aspects of CHH correlated positively with being seen at a specialized center (R = 0.40, p < 0.001) while having a provider who understood patients’ feelings about having CHH was positively correlated with having been referred for psychological counseling (R = 0.22, p < 0.05). The patient perspectives identified in the survey suggest that while many CHH patients are able to find and obtain specialized care/consultation with expertise in handling the medical aspects of their care, the emotional and psychological aspects of CHH are underappreciated.

**Figure 3:**
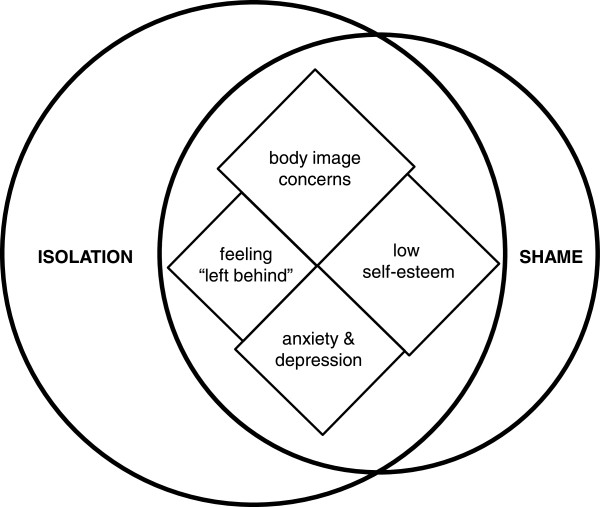
**Patient-reported challenges of CHH.** Patient-reported challenges represent targets for interventions. Focus group discussions revealed two dominant themes relating to feelings of isolation and shame (depicted by circles). These themes encompassed 4 consistent, inter-related psychosocial challenges related to CHH low self-esteem, body image concerns, feeling left behind by their lack of sexual development, and issues related to anxiety and depression (depicted by diamonds). The shapes are sized according to the frequency of patient comments and overlaps and connected shapes identify co-occurring themes.

Based on the survey findings we conducted three patient focus groups (n = 26 total participants, mean age 37 ± 13, range 18–66, median 36 years) to explore the challenges patients face in living with CHH and identify facilitators of adaptive coping strategies that could be leveraged for potential online interventions. What emerged from these discussions were consistent, pervasive psychological and emotional issues related to feelings of isolation and shame (Figure 3). The mean age at diagnosis was 18 ± 5 years (range 10–27, median 17 years) and spending teenage years and young adulthood in a prepubertal body was emotionally traumatic for many. Indeed, many patients commented on their frustration with what they perceived to be a late diagnosis or a delay in initiating treatment as well as significant concerns related to underdeveloped genitalia. These experiences were linked to 4 coherent themes: i) body image concerns, ii) low self-esteem, iii) anxiety/depression, and iv) a sense of being left-behind as their peers developed into adult bodies (and adult roles) (Figure 3, Table 2). Themes were frequent and their co-occurrence within and across groups suggest that a saturation point had been reached. These unmet psychosocial health needs indicate the need for psychological support and represent targets for much-needed intervention. Notably, differences were observed across different national health systems. For instance, patients with nationalized health systems reported better continuity in care (i.e. better transitions) and fewer gaps when changing healthcare providers. In contrast, patients in individual payer systems reported significant transition problems as well as medication coverage issues both of which undermined adherence to treatment.

**Table 2:** Table of themes emerging from focus group discussions, representative quotes, and targets for interventions

**Theme**	**Representative quotes**	**Targets for interventions**
***Patient-Reported Challenges***		
**Isolation**	"I was on the outside of any social group or gathering and on the inside, I was very alone and depressed and isolated and very frightened"	**connecting with others & peer-to-peer support**
	"I realized that something was “wrong” with me, and didn’t know what, and it looked like nobody knew. I more or less expected to have a unique disease which would have my name."	
	"I’ve pretty much been in exile for the past ten years"	
**Shame**		
i. Body image	(the hardest part of CHH) "The body image problems really… I will have that until the day I die"	
	"I can’t get undressed… I haven’t been swimming in many years. I can’t even pull my sleeves up… I can’t sit there like you with your sleeves rolled up…I’ve got to keep covered."	
ii. ↓ Self-esteem	"I have a tough time engaging with people, talking with people. Usually, when I am out in public I tend to look down at the ground, because you know… I feel so ashamed"	**cognitive- behavioral interventions**
	"I always thought there was a big spotlight on me all the time. Just… self-conscious… absolutely. I got a lot of bullying and being pushed around and abused when I was young"	
iii. Left behind	“I’m 40 years old and should be at the prime of my career and I don’t see myself…I don’t know, it’s a psychological barrier that I can’t progress, I’m stuck "	
	"For Kallmann’s (CHH), it’s sort of a crucial sort of time you know, we say the psychological and emotional things are equally as big as the medical forms of treatment for it. That big, big thing (absent puberty) carries on for the rest of your life"	
iv. Depression & anxiety	"I have depression, definitely. I have noticed a common denominator with depression and self medication… myself included"	
	"…and my family, they weren’t supportive. They didn’t help me. So, I got depressed and I tried to take my own life and then um…I left to seek help on my own"	
***Negative Healthcare Interactions***		
Lack of information	"The professor (doctor) who diagnosed my condition didn't even touch on the psychological side of things"	**online information & resources**
	"For the first few years after it (CHH) was mentioned to me, there was nothing at all coming back from the doctors. They didn’t really tell me what I was being treated for. So it was kind of…they diagnosed me but didn’t tell me and anything"	
	"The doctors weren’t… it was just nothing. There was no real after-care at all after the diagnosis. It was just ‘you need to take these injections’ and that was that…you know, for the rest of your life. There was no kind of…nothing. It was a lack of communication really"	
Disregard for feelings	"I felt like I wasn’t considered intelligent enough to understand what I was being treated for. It was like ‘oh, you won’t understand, it's complicated’. That’s it…yeah, it’s not nice to be made to feel that way."	**promoting patient-centered approaches & developing a "talking sheet" to initiate discussions with providers**
	"Somebody once said something to me, actually it was an endocrinologist, and I said ‘but I’m not normal’ you know? And this was several years ago, and I think he was trying to say ‘look, everything will be alright, keep taking your medication and all the rest of it’ and I said ‘I don’t know what normal is’…and that didn’t seem to faze him at all. I sat here saying to this professional , educated, intelligent man - he’s a professor - and I was saying ‘I don’t know what normal is’ and he didn’t respond. Nothing…he didn’t even look at me."	
	"There’s no sense from anyone… about them trying to understand or even that it crosses their mind that you are going through anything. You know, that it’s painful. They are just ‘Mr. fix-it’ - give you a prescription and you are gone"	
Lack of shared decision-making	"He (doctor) didn’t give me any treatment options. He just said 'take this gel'. We didn’t discuss what was the best treatment. I don’t know if it was just the physician that I went to…maybe there are better ones out there who would have given me the option(s)"	
	"The first doctor I saw he said just take these and you’ll be ok"	
Discordant expectations for treatment outcome	"No one really explained to me…I thought that if I took the testosterone…I didn’t understand that…I thought that if I just took the testosterone that I would go through puberty and I would be normal."	**online anticipatory guidance information**
	"I said no, I can’t smell a thing and he said, 'Ah, you’ve got Kallmann's (CHH)!' I thought wow that’s great, give me the injections and I can smell the roses and all that and well of course it didn’t happen. My sense of smell never came."	

The first column identifies thematic elements from the focus group discussions across two topic areas: patient-reported challenges and negative healthcare interactions. The middle column presents representative quotes for the emergent themes. The third column lists the related targets for interventions to address the unmet health and informational needs of the CHH men. Quotes referencing the term "Kallmann's" refers to Kallmann syndrome - the association of CHH with the inability to smell (anosmia).

### CHH patients have unmet health information needs

During focus group discussions, participants indicated that healthcare providers (physicians, nurses and pharmacists) played both positive and negative roles in helping them adapt to living with CHH (Table 2). Providers who were not forthcoming with information about CHH, disregarded patient concerns/feelings, did not set appropriate expectations for treatment outcomes, and did not engage patients in decisions, were sources of frustration for patients and undermined patients’ sense of wellbeing. Healthcare professionals could also be powerful promoters of adaptive coping. Patients reported feeling supported by providers who expressed empathy, facilitated self-management techniques (i.e. self-injecting medications) and involved them in decision making. As such, efforts to promote patient participation in treatment decisions and providing patient information and anticipatory guidance in lay language appear to be avenues for meeting patients’ informational needs, and potentially improving patient satisfaction with care and adherence.

### CHH patients rely on the internet for CHH information and peer-to-peer support

The survey participants were active users of the internet for issues related to their condition. Nearly all participants (101/105, 96%) stated they had sought information from online sources (i.e. Wikipedia). This may be related to the fact that the manifestations of CHH (sexual immaturity and infertility) are very private and personal matters and thus may contribute the high rate of anonymous information seeking that the internet provides. Less than half of survey respondents (49/105, 47%) used the medical literature as a resource for finding out about CHH. This was interesting as these men have relatively high levels of education (Table 1), yet preferred to seek information from other online resources, paralleling the increase in such practices among physicians [44]. Because the number of years of formal education may not be the best predictor of facility with medical information, we also used a previously validated self-report question “How confident are you filling out medical forms by yourself?” to screen those participants with inadequate healthcare literacy [33,34]. By this standard, all but one of participants had adequate healthcare literacy. Therefore, despite adequate education, appropriate healthcare literacy and access to the literature (i.e. PubMed Central [45]), it seems that patients are much more likely to seek out information from online sources that are not part of the medical literature. Therefore, providing expert-reviewed information in lay language online could be an effective way to disseminate CHH-related health information to these dispersed patients.

The survey also revealed that patients seek information from healthcare providers and the online community in equal proportions (74/105, 70% and 81/105, 77% respectively). Further, when asked to rate the importance of healthcare professionals, online community and the internet as sources for information, all rated similarly (p = 0.58). These data support the notion that for CHH patients, information from healthcare providers and members of the online community who share their condition are equally important and complementary. The importance of connecting with other patients via the online community was echoed in the patient focus groups. Participants stated that connecting with other patients online and finding out they were not alone was an important aspect of coming to terms with CHH, finding meaning in their condition, and being able to not let CHH dominate their life. Further, face-to-face encounters with other patients were often regarded as a pivotal and life-changing event (Table 3).

**Table 3:** Patient-reported facilitators of coping

**Theme**	**Representative quotes**	**Targets for interventions**
**Meeting others & online support**	“Though I have a loving family I had spent most of my life feeling depressed, confused, lonely, alone, isolated, and frequently in despair. In just a couple of hours at the meeting 3 years ago… those feelings decreased. For the first time ever I was with a group of people with whom I felt normal and at ease, valued and respected. This has made a tremendous difference in my life."	**Online peer-to-peer support**
	"It wasn’t until I found other people like (patient community leader) that I kind of filled in the blanks a bit. It was quite isolating for me and I had no one to talk to and I felt like I was the only person in the world to have this problem"	
	"I’ve really felt alone. So I went online, on Facebook just to see if there was something or a meeting and I found this group and it was the best thing I ever did because I found out there are other people and I thought I was alone. But I found others… which is really good"	
	"Going to my first Kallmann (CHH) meeting about 6 years ago… and until then, I was totally in the dark. And when I met up with fellow patients, I realized I’m not on my own"	
**Coming to terms with CHH**	“Get over it, take matters into your own hands… you will get help. All you have to do is ask for it. (To the moderator) in your research, you are searching for ways that would help me… a toolkit, a fact sheet ‘living with Kallmann (CHH)’ that kind of thing…it also involves emotional things. If you can give that to a doctor so then that is the trigger to start a conversation. Then you can take matters into your own hands”.	**Patient empowerment**
	"So it was a change in mentality, being taunted as a child then I realized that I had to do certain things to get my life back"	
	"It was just the things that I would have to find out about myself that no one could tell me… and like I said, (patient community leader) has helped… Both on a personal level and for the research into the condition itself. But, being able to live with it is a very personal thing that I think you need to find out for yourself…and I think I’m coming along pretty nicely… I’m not letting it rule my life quite so much as I used to."	
**Positive healthcare interactions**	"All the medical professionals I have been working with…I’ve been very lucky because they have always been very helpful and considerate about it. So, I guess I have been fortunate that way"	**Promoting patient-centered approaches & developing a "talking sheet" to initiate discussions with providers**
	"Definitely, self-injecting (helped). Learning about the syndrome made me feel that Kallmann (CHH) is not such a big deal. But in my case, it was not enough… and the psychotherapy aspect helped a lot"	
	"I first heard about Kallmann's (CHH) at 25 when I moved in a new city and changed endocrinologists. That made a huge difference, suddenly she made it sounds like it was not such a big deal. In my experience female doctors are more easy to talk with, tend to ask more about how it works in the everyday life, and involve us in our prescriptions"	

The first column identifies the central emergent themes from focus group discussions related to effective coping. The middle column presents representative quotes. The third column identifies potential avenues for interventions to promote coping and resilience among CHH men. *Quotes referencing the term "Kallmann's" refers to Kallmann syndrome - the association of CHH with the inability to smell (anosmia).

The data from the present study are in line with the findings of a 2011 report from the Pew Foundation that identified patients with rare diseases as the most likely group to draw upon online peer support network, even more so than those with other, more common chronic health conditions [6]. Patient knowledge is different from that of professional healthcare providers. Patient expertise grows from a personal day-to-day experience of living with a condition and as such, patients can provide critical informational support for coping and managing one’s health [46]. These complementary realms of expertise present an opportunity for collaborative efforts for health promotion and improving quality of life for patients dealing with chronic conditions [47-49]. Connecting with other patients is critical for CHH because it can diminish the isolation of living with a rare condition. Further, such peer-to-peer support may hold important promise for very practical and concrete benefits such as promoting adherence to treatment and continuity with healthcare and encouraging peers to seek mental health services when needed.

### CHH patients are receptive to online interventions

Focus group participants were unanimously open to and in support of utilizing online approaches to help develop coping and self-management skills. The only concern raised regarding this was the issue of confidentiality that perhaps is not surprising given the very private nature of the sexual immaturity and infertility of CHH. This mixed-methods study identified a variety of unmet health needs of CHH men. Importantly, the focus groups revealed positive examples of patients overcoming challenges, finding meaning in their condition and becoming empowered, activated patients (Table 3). As these men are active internet users and receptive to the idea of online interventions, we propose several patient-centered approaches for health promotion in these dispersed patients.

### Developing online interventions to address the needs of CHH patients

Interventions addressing the identified shortfalls in care for CHH patients are intended to enhance wellness and improve coping and quality of life for these individuals. This is the fundamental objective of health promotion – the process of enabling people to overcome health challenges and increase control over their situation to achieve greater health [50]. Indeed, the Health Promotion Model [51] has been used as a model to develop patient-centered approaches to activate and empower patients for self-management [52] and thus seems fit the needs of CHH patients. This approach acknowledges patient expertise and dramatically reframes patient involvement as well as the role of the healthcare professionals to redefine successful health outcomes [53]. Herein we identify three main targets for health promoting interventions (Tables 2 and 3).

First, CHH patients have unmet informational needs related to their condition. Given their active internet use, it seems logical to launch web-based information resources for patients and providers alike. For patients, this could include information in lay language about CHH, treatment options, and anticipatory guidance related to treatment (i.e. what to expect from treatment and in what timeframe). Further, we propose to develop materials (i.e. fact sheet of discussion points) for healthcare professionals, written by expert clinicians and available online for patients to use in order to initiate discussions with their healthcare provider on the psychological and emotional aspects of the condition and to promote shared decision-making. These materials would help contribute to create a virtual online empowerment toolkit for patients to learn about their condition, find expert care, and become empowered to take control of their health. Further, these resources could be provided in multiple languages via the European network studying GnRH deficiency [30] encompassing patient information in lay language, listings of specialized referral centers, genetic testing resources, clinical trial listings, and web-based platforms for contacting expert clinicians as well as links with peer-to-peer support.

Second, enhancing online peer-to-peer support to diminish the sense of isolation, share coping strategies and encourage peers to seek mental health services when needed to address issues of anxiety/depression. The synergy of online connectivity and patient expertise facilitates crowdsourcing for the rare disease community. Crowdsourcing is the process of tapping into the collective knowledge and problem solving abilities of a group (such as patients with a shared medical condition) to generate ideas and solutions [54]. Importantly, online patient communities are not intended to replace existing healthcare infrastructures, rather their expertise differs from healthcare professionals and can be valuable for peer-to-peer support and learning how to deal with issues on a daily basis [46]. Further, this pool of knowledge/experience not only impacts health management, but is also beginning to change approaches to research [55].

Third, to help patients address their feelings of shame, body image concerns and low self-esteem there is an opportunity to utilize online cognitive-behavioral interventions. Indeed, online education combined with peer-to-peer coaching has been successful in enhancing self-management and adherence to treatment for bipolar disorder and diabetes [56,57]. A similar approach combining online patient education, cognitive behavioral interventions, and peer coaching could be adapted to address the specific body-image and self-esteem issues related to CHH. Further, technology may enable contact with otherwise difficult to reach patients and potentially surmount barriers that exist for face-to-face mental health treatment.

### Limitations

Like other studies of this kind, this needs assessment has limitations. First, the inferences drawn from the survey findings could be strengthened by a larger sample size. Indeed, recruiting adequate numbers of rare disease patients can pose a challenge. This is why we utilized a web-based approach, yet this is not without bias as not everyone has internet access and is perhaps reflected in the fact that respondents were highly educated. Further, an online survey in English may introduce a potential Anglophone bias. Importantly, the longstanding critique of online research (access) is beginning to fade as the number of individuals lacking internet access rapidly shrinks. As of 2011, 73% of European Union households had internet access (2/3 of which have broadband). Further, more than half of Europeans use the internet daily and just one quarter (24%) have never used the internet [58] and many developing countries have “leapfrogged” straight into mobile handsets for multimodal communication and connectivity [59].

Second, there are potential biases in the sampling of subjects. Participants were recruited via expert clinicians as well as among patient support groups. As medical record review was not part of this study, we recontacted 40% of respondents to confirm diagnosis. As such, we cannot be sure that every respondent met all hormonal and clinical criteria of a CHH diagnosis. However, the fact that the findings of the survey were mirrored in the focus groups (where all participants had confirmed CHH diagnosis) contributes to the validity of the study. Additionally, engaging patients in the study development and conducting an internet survey introduces sources of potential bias as these patients are the ones who seek out forums and web-based support. Similarly, those participating in patient support groups could represent men who are struggling most to deal with their condition and thus may overestimate the difficulties experienced by men with CHH. However, these issues may be less of a concern for such a needs assessment as the intent was precisely to identify a specific cohort of patients to explore their situation and perspectives in depth. While not a random sampling of patients, targeting internet users was necessary to assess the acceptability of delivering web-based interventions to patients who use the internet as a resource for health information and support. In addition, the anonymity of online information can be an asset for eliciting information that patients may not feel comfortable expressing in face-to-face encounters with healthcare providers [60].

Third, the evaluation of adherence is often debated as there is no gold standard definition, no clear consensus on what is an acceptable level of adherence and self-report is subjective and an inherently flawed method. An alternative approach would be to perform retrospective chart reviews and comb pharmacy refill information, but these are beyond the scope of this needs assessment study. Rather, this survey was intended to capture a global picture adherence patterns in this patient population. The rationale for using a self-report measure was based on the fact that CHH is a chronic condition and as such, patients are responsible for the bulk of their care. Therefore, understanding patient perceptions and perspectives is a critical first step in developing patient-centered approaches to activate individuals and enhance self-management of their chronic condition.

## Conclusions

Patients with CHH often have long gaps in care and struggle with significant psychosocial sequelae that are often unrecognized by the healthcare community. These patients are active internet users who draw on social media and online communities for support and to complement the information received from healthcare professionals. Patients are receptive to online interventions aimed at addressing their unmet needs. Peer-to-peer support can help enhance coping and patients should be encouraged to utilize these online communities. Drawing upon patient expertise and developing partnerships with online patient communities may provide new opportunities for health promotion and improved quality of life for these patients.

## Abbreviations

CHH: Congenital hypogonadotropic hypogonadism; KS: Kallmann syndrome; COST: European cooperation in science and technology.

## Competing interests

The authors have no financial or non-financial competing interests to declare.

## Authors’ contributions

AD conceived the study and participated in study design, conduct, analyses and drafted the manuscript. RQ participated in the coordination and conduct of the study. NP participated in study design and analysis. DM participated in study design, and analysis. All authors read and approved the final manuscript.

## Supplementary Material

Additional file 1 Supplemental Materials summarizing the online survey results and Figure S1 depicting longest duration without healthcare. Click here for file
