# Supplementary material for: Identifying the unmet health needs of patients with congenital hypogonadotropic hypogonadism using a web-based needs assessment: implications for online interventions and peer-to-peer support
Source: Orphanet J Rare Dis. 2014 Jun 11;9:83. doi: 10.1186/1750-1172-9-83 (PMC4059885; doi:10.1186/1750-1172-9-83)
Supplement: Additional file 1 — Supplemental Materials summarizing the online survey results and Figure S1 depicting longest duration without healthcare. [file 1750-1172-9-83-S1.pdf]

SUPPLEMENTAL MATERIALS: Identifying the unmet health needs of patients with congenital hypogonadotropic hypogonadism using a web-based needs assessment: Implications for online interventions and peer-to-peer support

1.Summary of online survey results

2. Supplemental Figure S1: Patient-reported longest duration without healthcare among CHH men.

1.Summary of online survey results

1A. Sociodemographic information

Age: [open ended response]

- 19-29 yrs: 32/105 (30%)
- 30-39 yrs: 39/105 (37%)
- 40-49 yrs: 19/105 (18%)
- 50-59 yrs: 11/105 (10%)
- 60+ yrs: 4/105 (4%)

Relationship status: [multiple choice]

- never been in a relationship: 24/104 (23%)
- single: 25/104 (24%)
- in a relationship: 16/104 (15%)
- married: 38/104 (36%)
- divorced: 1/104 (1%)

Religion: [open-ended question question]

- Christian (protestant & evangelical): 25/90 (28%)
- Roman Catholicism: 17/90 (19%)
- Orthodox Christianity: 3/90 (3%)
  (Eastern & Russian)
- Islam: 5/90 (6%)
- Judiaism: 2/90 (2%)
- Buddhism: 1/90 (1%)
- Hinduism: 1/90 (1%)
- Other: 10/90 (11%)
  (Paganism, Mormonism, Jehovah's Witness, secular humanist, personal faith)
- Atheism: 10/90 (11%)
- None: 16/90 (18%)

Highest level of education: [multiple choice]

- elementary: 0/105 (0%)
- high school/vocational: 36/105 (34%)
- university: 38/105 (36%)
- post-graduate: 31/105 (30%)

Employment: [multiple choice]

student: 9/104 (9%)
working part-time: 9/104 (9%)
working full-time: 70/104 (67%)
retired: 6/104 (6%)
unemployed: 10/104 (10%)

Type of employment: [open ended question - responses included]

Business & Finance<br>Business analyst/consultant<br>Economist<br>Finance	*Teaching & Education*<br>Teacher<br>Professor<br>School administrator
Law & Legal Services<br>Attorney<br>Legal assistant<br>Legislative consultant	*Psychology & social Work*<br>School Counselor<br>Counselor/mediator
Healthcare & Science<br>Dentist<br>Physician<br>Nurse<br>Pharmacist<br>Ambulance staff<br>Patient care assistant<br>Translation services<br>Biomedical scientist<br>Chemist/Lab Technician<br>Hospital finance/administration	*Engineering & Information Technology (IT)*<br>Engineer<br>Electrical Engineer<br>Applications engineer<br>Network Engineer<br>Computer systems management<br>Software developer<br>Computer programming<br>IT technician
Service & retail<br>Clerk<br>Sales/Retail<br>Personal trainer<br>Call center/customer services	*Management & supervisors*<br>Manager - small business<br>Manager – manufacturing<br>warehouse supervisor<br>Purchasing manager
Laborer & Manufacturing<br>Construction<br>Lawn care<br>Airline baggage<br>Warehouse<br>Milling machine operator<br>Quality control technician	*Transportation*<br>Public transportation driver<br>Limousine Service<br>Truck driver
Civil Service<br>civil servant<br>Postal worker<br>Juvenile services	*Arts & Entertainment*<br>Composer<br>Performing arts<br>Graphic design
Religious clergy<br>clergy<br>Buddhist monk	

1B. Healthcare literacy

- *How confident are you filling out medical forms by yourself?*
  Extremely: 45/105 (43%)
  quite a bit: 34/105 (32%)
  somewhat: 25/105 (24%)
  a little bit: 1/105 (1%)
  not at all: 0/105 (0%)

1C. Health information seeking patterns:

- *Where have you searched for information to learn about CHH? (check all that apply)*
  internet (i.e. Wikipedia): 101/105 (96%)
  online community (social media/chat): 81/105 (77%)
  healthcare professionals: 74/105 (70%)
  medical literature: 49/105 (47%)
  family/friends: 12/105 (11%)
  other: 0/105 (0%)

- *From your experience, please rank the 3 most important sources:*
  healthcare professionals: weighted score 166
  internet (i.e. widkipedia): weighted score 150
  online community (social media/chat): weighted score 146
  medical literature: weighted score 93
  family/friends: weighted score 18

1D. Interactions with healthcare system/providers

- *Within the healthcare system, have you ever experienced discrimination because of CHH?*
  No: 89/105 (85%)
  Yes: 16/105 (15%)

- *Have you ever had a consultation or received treatment at an academic health center (such as a teaching hospital or research center)?*
  No: 51/105 (49%)
  Yes: 54/105 (51%)

- *Is there a healthcare provider (i.e. doctor or nurse) who you feel really understands the medical aspects of your CHH?*
  No: 35/105 (33%)
  Yes: 70/105 (67%)

- *Is there a healthcare provider (i.e. doctor or nurse) who you feel understands your feelings about having CHH?*
  No: 65/104 (62.5%)
  Yes: 39/104 (37.5%)

- *Has a healthcare provider (either general practitioner or specialist) ever offered you counseling or a referral for professional counseling?*
  No: 79/105 (75%)
  Yes: 26/105 (25%)

1E. Diagnosis, treatment & adherence

- *At what age were you diagnosed with CHH?* [open ended question]
  mean±SD: 18±6 years
  range: neonatal – 32 years
  median: 18 years

- *Lifetime duration of treatment* [calculated from start of treatment]
  mean±SD: 18±12 years
  range: 0 – 46 years
  median: 16 years

- *What treatments have you had for CHH? (check all that apply)*
  testosterone (injections, pellets, patches, or gel): 99/105
  gonadotropin/fertility injections: 37/105
  none: 3/105 (two recently diagnosed patients)

- *What is the longest period of time that you have gone without medical care (not under the care of a doctor or healthcare provider for your CHH):* [open-ended]
  Never: 24/93 (26%)
  <6 months: 27/93 (29%)
  6-12 months: 7/93 (8%)
  13-24 months: 15/93 (16%)
  25-36 months: 8/93 (9%)
  36+ months: 12/93 (12%)

- *What is the longest period of time that you have been off your CHH medication (not at the instruction of your doctor or healthcare provider):* [open-ended]
  never: 28/87 (32%)
  <6 months: 20/87 (23%)
  6-12 months: 3/87 (3%)
  13-24 months: 17/87 (20%)
  25-36 months: 6/87 (7%)
  36+ months: 13/87 (15%)

Figure S1. Patient-reported longest duration without healthcare among CHH men.

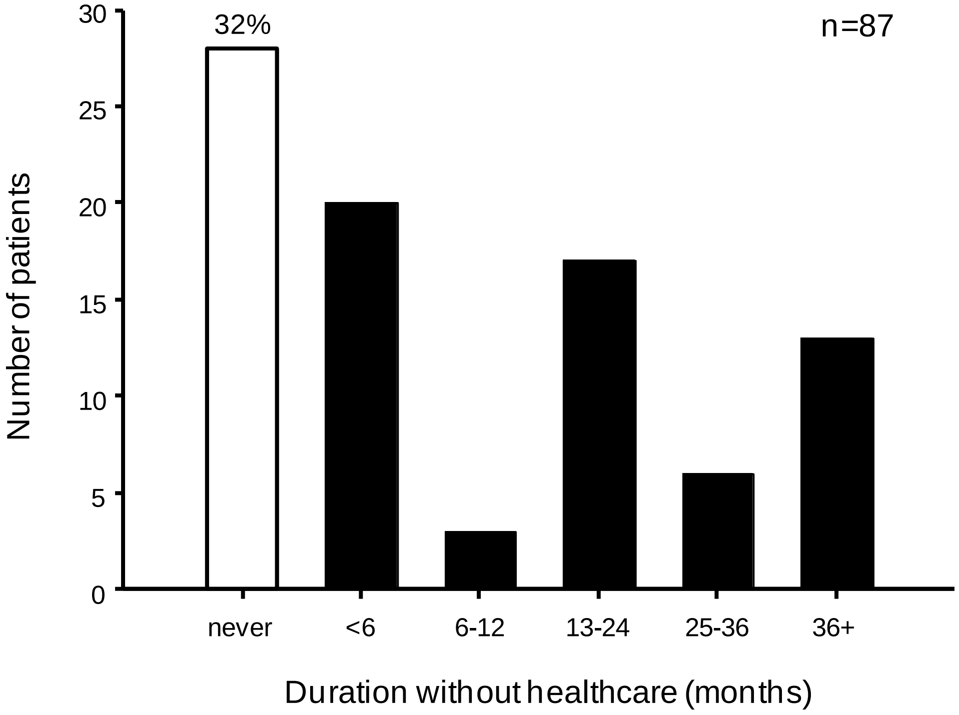

Healthcare continuity as assessed by patient-reported longest period without contact with a healthcare professional (n=87). In total, 32% of CHH men reported never having a break in their continuity of healthcare while 41% had gaps in care exceeding 1 year.
